# Sex Differences in Animal Models of Sodium-Valproate-Induced Autism in Postnatal BALB/c Mice: Whole-Brain Histoarchitecture and 5-HT2A Receptor Biomarker Evidence

**DOI:** 10.3390/biology11010079

**Published:** 2022-01-05

**Authors:** Balaji Gouda, Sukesh Narayan Sinha, Meram Chalamaiah, Validandi Vakdevi, Patangay Shashikala, Bantal Veeresh, Venkata Mullapudi Surekha, Vasudev Kasturi, Naveen Kumar Boiroju

**Affiliations:** 1 Division of Food Safety, Indian Council of Medical Research, National Institute of Nutrition, Jamai-Osmania, Hyderabad 500007, India; balajiroy4@gmail.com (B.G.); vaagdevi@gmail.com (V.V.); vasudevnin@gmail.com (V.K.); 2 Drug Safety Division, Indian Council of Medical Research, National Institute of Nutrition, Jamai-Osmania, Hyderabad 500007, India; chalamaiah.m@gmail.com; 3 Department of Pharmacy, University College of Technology, Osmania University, Hyderabad 500027, India; shashikala_patangay@yahoo.co.in; 4 Department of Pharmacology, G. Pulla Reddy College of Pharmacy, Osmania University, Hyderabad 500028, India; veereshsetty@gmail.com; 5 Division of Pathology and Microbiology, Indian Council of Medical Research, National Institute of Nutrition, Jamai-Osmania, Hyderabad 500007, India; surekha_mv@yahoo.com; 6 Division of Biostatistics, Indian Council of Medical Research, National Institute of Nutrition, Tarnaka, Hyderabad 500007, India; Boiroju.nk@icmr.gov.in

**Keywords:** autism spectrum disorder, BALB/c mice, valproic acid, brain histology, scanning electron microscopy, 5-HT2A receptor protein, postnatal day

## Abstract

**Simple Summary:**

Valproic acid (VPA) is a well-known antiepileptic medication and mood stabiliser that is frequently prescribed for the treatment of epilepsy, particularly in children, and has proven human teratogenic activity. VPA inhibits histone deacetylase, which causes teratogenicity and cell toxicity. VPA-induced autism in rodents during the pre- and postnatal periods has shown the development of an autism-like phenotype. In mice, the 14th postnatal day is thought to correspond to the third trimester of human development; it is an important period in which neuronal migration, differentiation, myelination, synaptogenesis and gliogenesis occur in the cerebellum, striatum and hippocampus. Therefore, we exposed postnatal day 14 (PND 14) mice to VPA, which resulted in autistic-like behaviours manifested as reduced social interaction, increased repetitive stereotyped behaviour and anxiety, cognitive dysfunction, lowered sensitivity to pain and neurodevelopmental delay. BALB/c mice were used in this work because they are less reactive to social contact in VPA-induced autism than many other inbred mouse strains, such as C57/129 mice. In humans, two to three times more men are affected by autism spectrum disorder (ASD) than women, and, for this reason, the current study compares the histopathological changes and 5-hydroxy-tryptamine 2A (5-HT2A) receptor protein expression in the brain tissue of male and female animals with VPA-induced autism.

**Abstract:**

Autism spectrum disorder (ASD) is characterised by problems with social interaction, verbal and nonverbal communication and repetitive behaviour. In mice, the 14th postnatal day is believed to correspond to the third trimester of human embryonic development and is considered a vital period for central nervous system development. It has been shown that ASD affects 2 to 3 times more male than female individuals. In the present study, ASD was induced in 14 postnatal day (PND) BALB/c mice using valproic acid (VPA). VPA administration brought about substantial differences in the histoarchitecture of the brain in both male and female mice, linked to behavioural deficits. We observed that both male and female mice showed similar morphological changes in the prefrontal cortex, hippocampus and Purkinje cells. We also observed hair loss from PND 17 to 25, which was again similar between male and female mice. However, there were higher rates of change in the cerebral cortex, frontal cortex and temporal lobe and hippocampus in VPA-treated male animals. With respect to the cerebellum, we did not observe any alterations by haematoxylin and eosin (H&E) staining, but detailed morphological observation using scanning electron microscopy (SEM) showed a higher rate of phenotype changes in VPA-treated male animals. Moreover, 5-HT2A receptor protein levels were upregulated in the cerebral cortex, hippocampus and Purkinje cells in VPA-treated male mice compared with control animals and VPA-treated female mice, as shown by immunohistochemical analysis. Based on all these findings, we conclude that male animals are more susceptible to VPA-induced ASD than females.

## 1. Introduction

Autism spectrum disorder (ASD) is a group of neurodevelopmental disorders characterised by early-onset social communication difficulties and the emergence of extremely constrained and repetitive behaviour patterns [1]. ASD affects roughly 20 out of every 10,000 children, and early symptoms of the illness can be detected in children as young as 1 to 3 years old [2]. Additionally, autistic people suffer from mental retardation and epilepsy [3]. The causes of autism are unknown, but multiple factors lead to its development, such as genetic aberrations, environmental insults and social influences [4,5]. While it is generally suspected that genetic factors are involved in the development of ASD, they are not responsible for all cases [6]. In addition, increased oxidative stress, hyperserotonaemia and loss of Purkinje cell integrity in the cerebellum may lead to autism [7]. Environmental factors such as prenatal viral infections [6] and chemical and drug exposure in the mother may also increase the risk of ASD in the offspring [8]. Chemicals such as ethanol, mercury, thalidomide and misoprostol trigger the production of reactive oxygen species (ROS), responsible for cerebellar, limbic and brain development deficits [9].

VPA is a drug widely used in the treatment of various neurological disorders, such as epilepsy and resistant depression, for migraine prophylaxis, and as an antiepileptic and mood-stabilising agent [3]. VPA-induced animal models are widely used by researchers worldwide for the investigation of autism, because of its property of effecting abnormality in the neurodevelopment of the cerebellum and other areas of the brain, with disturbances in synaptic integration [10].

Induction of autism by VPA in rodents during pre- and postnatal periods has shown the development of autism-like neurobehavioural defects comparable to the motor and cognitive disorders, nociceptive response, locomotion, anxiety and prolonged deficits in the maturation of social behaviour [11,12,13] of those observed in autism patients [14,15,16]. For mice, the 14th postnatal day is believed to correspond to third trimester of human development [15,17] and it is considered to be an essential phase during which neuronal migration, differentiation, myelination, synaptogenesis and gliogenesis take place in the cerebellum, striatum and hippocampus [17].

Even though many studies on VPA-induced autism have been done with male animals [3,18], few studies have been performed using both male and female animals [9,11]. Previous studies reported that ASD affects 2 to 3 times more men than women [12]. The main reason for choosing male animals for ASD research is due to the higher incidence of the disease in men than in women, as well as the difficulties that arise during the oestrous cycle in female animals, especially at the time of behavioural analysis [19]. Kazlauskas et al. [19] reported that both male and female offspring of mice with VPA-induced autism showed disturbances in postnatal behaviour.

Previous studies have demonstrated that VPA treatment of rodents on PND 14 resulted in intrusions and neurodevelopmental regressions, reflected by many behavioural retardations and the degeneration of cells in the cerebellum and hippocampus [9,15]. Clinical evidence, neuroimaging, genetic disorders and post-mortem findings suggest that cerebellar dysfunction may play a vital role in the aetiology of ASD [20]. The cerebellum is one of the most consistent diagnosis sites of ASD, in both humans and animals [20]. The cerebellar cortex was found to be abnormal in 26 mouse models of ASD [21], and cerebellar atrophy is one of the characteristics of ASD animal models induced by VPA [22]. There is constant communication between the cerebellum and cerebral cortex, with instructions from higher levels regarding the brain’s intentions by processing in the cerebellar cortex and sending messages to the cerebral motor cortex or inducing voluntary muscle contractions.

The 5-HT2A receptor is a G-protein-coupled receptor (GPCR) on the cell surface that belongs to the serotonin receptor family. The 5-HT2A receptors are distributed throughout the brain in mammals, with the highest density found in the cortex, frontal cortex, limbic system and cerebellum [23,24]. Moreover, 5-HT2A receptors play a crucial role in specific CNS disorders, such as schizophrenia, depression, epilepsy, obsessive-compulsive disorder and ASD, according to pharmacological and genetic investigations [23,25].

The aim of this investigation was to compare the histopathological changes and 5-HT2A receptor protein expression in the brain tissue of male and female animals with VPA-induced autism. Additionally, we assessed the histopathological abnormalities and 5-HT2A receptor protein expression associated with behavioural deficits.

## 2. Material and Methods

### 2.1. Chemicals

Sodium valproate (VPA) was purchased from Sigma-Aldrich, St. Louis, MO, USA. Normal saline and water for injection were purchased from Aculife Healthcare Pvt. Ltd. (Sachana, Guarat, India). Formalin was purchased from SD Fine Chem Limited (Chennai, Tamil Nadu, India).

### 2.2. Animals

Male and female BALB/c mice were put together in plastic cages with conducive environmental conditions: controlled temperature (25 ± 2 °C) and humidity (55%) and 12 h light/dark cycle. Food was kept within reach and water was freely available. Physical assessment of the female animals was conducted every day; in the case of vaginal plug appearance in any animal, it was considered as embryonic day (ED) 0, and the date of birth was registered as PND 0. All experimental protocols for the management and supervision of animal experiments were carried out in compliance with CPCSEA guidelines. The Institutional Animal Ethics Committee reviewed and approved this study (ICMR-NIN/IAEC/02/007/2019, ICMR–NIN Animal Facility, National Institute of Nutrition, Hyderabad, India).

### 2.3. Experimental Design

**Figure 1:**
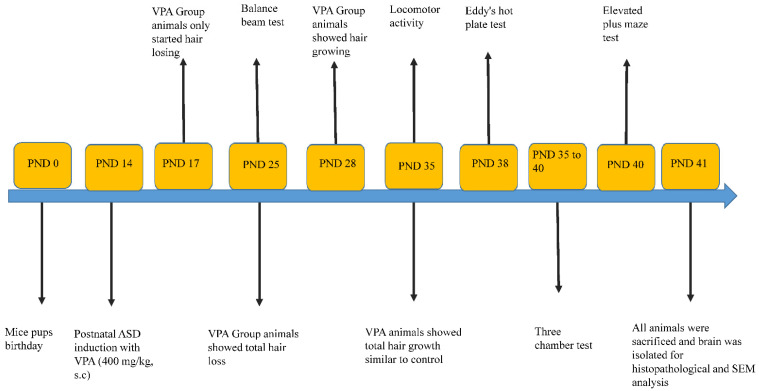
A clear timeline picture of study design including VPA injection, behavioral tests and, sample collection for histopathological, immunohistological and SEM analysis.

Two groups of animals were designed, each group comprising 12 animals (6M and 6F) from 6 different litters. VPA solution was prepared in saline at a concentration of 80 mg/mL, and the dosage was determined according to body weight. The first group, considered the control, was given saline subcutaneously on PND 14, followed by sterilised water by oral administration until PND 40. Group 2, considered the VPA-treated group, received a single subcutaneous dose of VPA (400 mg/kg) on PND 14, followed by sterilised water by oral administration until PND 40. All behavioural tests were conducted from PND 25 to 40. All animals were sacrificed on PND 41, and brains were isolated for histopathological examinations (Figure 1) [9,12,14,15,26].

### 2.4. Behavioural Analysis

#### 2.4.1. Balance Beam

To evaluate motor coordination disability, a balance beam test was conducted on all animals, as described in earlier reports [14,27]; this test was done on PND 25. The instrument consisted of a beam 80 cm long and 10 mm wide, elevated to a height of 45 cm from the ground, with cushions beneath to protect the animals if they fell. We kept the experimental room completely dark and illuminated one side of the beam with a 60 W electric bulb, and the other side led to a dark 15 cm^3^ box with cushioning material from the animal’s home cage so as to make the surroundings familiar. The experiment was conducted on all animals for 3 consecutive days, considering the first 2 days as training and the third day as testing. On the day of the test, we captured video of the animals crossing the beam on each 60 s trial, and the number of foot slips, distance covered (cm) and time taken (s) were recorded when the animal moved from the illuminated side to the dark box.

#### 2.4.2. Nociception

This test was conducted on PND 38 to evaluate the nociceptive response by monitoring the latency of paw licking and jumping in the animal, and was recorded by using a stop-watch. Each mouse was placed on an Eddy’s hot plate analgesiometer (Sisco Instruments, 8UA Jawahar Nagar, New Delhi, India) with an electrically heated surface, with the temperature maintained at 55 ± 0.5 °C. A cut-off time of 15 sec was maintained [9].

#### 2.4.3. Elevated plus Maze

The elevated plus maze test was conducted on PND 40; this test was used to evaluate the animals’ anxiety level. The instrument consisted of 2 open and 2 closed arms 25 cm × 5 cm in size with closed opaque walls 15 cm high covering both closed arms, and the maze was constructed at a height of 55 cm from ground level. Each animal was placed facing one of the closed arms at the central 5 cm × 5 cm part of the instrument, and its behaviour was monitored for 5 min by recording the number of times the animal entered the open arm and how long it spent there [26].

#### 2.4.4. Actophotometer for Locomotor Activity

The locomotive activity test was conducted on each mouse separately on PND 35 using an actophotometer (Opto-Varimex-Minor, Columbus Instruments, Columbus, OH, USA). This instrument is designed with 3 infrared emitters on each side of the x- and y-axis and an equal number of receivers on the opposite side. Obstruction of the photo beams is considered a measure of locomotive activity, which was monitored by recording the number of beam breaks in a 5 min time interval [9].

#### 2.4.5. Assessment of Social Interaction

This test was conducted on PND 35–40 on a 3-chamber testing apparatus (57 cm × 36 cm × 30 cm) with passages from one chamber to another, as described earlier [13]. The experiment was conducted in 3 sessions. In the first session (5 min), the treated animal was placed in the middle chamber and was free to move in all directions; this is called the habituation phase. The other 2 chambers are called stranger and empty chambers. In the second session (10 min), the social ability phase, a stranger animal was placed in one of the side chambers, i.e., stranger chamber, with movement restricted. In the last social preference session phase of the experiment (10 min), a novel animal was placed in the empty chamber with movement restricted, similar to the stranger animal. In this phase, we considered the stranger and empty chambers as familiar and novel chambers, respectively. The treated mouse was free to explore all chambers throughout the experiment. The time spent by the animal in novel and stranger chambers was recorded for calculation of the sociability index (SI) and social preference index (SPI):

$$SI\;=\;\frac{Time\;spent\;in\;stranger\;chamber}{Time\;spent\;in\;empty\;chamber}$$

$$SPI\;=\frac{Time\;spent\;in\;novel\;chamber}{Time\;spent\;in\;familiar\;chamber}$$

### 2.5. Histopathology

All animals were sacrificed, and brain tissues were separated immediately and gently washed with saline so that they were free from mucus and debris, and were kept in 10% neutral formalin solution until the histopathological examination. Then, the tissues were processed for dehydration. The tissues were passed through successive series containing 30, 50, 70, 80, 90, 95% and absolute alcohol, and then cleaned in methyl benzoate and embedded in paraffin wax. Sections of 5 μm thickness were made using a rotary microtome. The sections were stained with Harris haematoxylin (Anatech Ltd., Thermo Fisher Scientific, Battle Creek, MI, USA) and counterstained with eosin dissolved in 95% alcohol. After dehydration and cleaning, the sections were mounted in Canada balsam. Photomicrographs of the section preparations were taken using Zeiss light microscope equipment [28].

### 2.6. Scanning Electron Microscope Analysis

Samples were fixed in cold 2.5% glutaraldehyde 25% (batch no. 62930, TAAB Laboratories Equipment Ltd., Aldermaston, UK) in 0.2 M sodium cacodylate buffer (pH 7.2; M. formula C_2_H_6_ASNAO_2_·3H_2_O, Sigma, St. Louis, MO, USA) in the fixative solution overnight at 4 °C for complete fixation.

The samples were washed with a working buffer (0.1M SCB) 3 times with 20 min intervals to remove the excess fixative.

All samples were dehydrated in ascending order of ethanol (ethyl alcohol 100%; Hayman Group Ltd., Essex, UK F204325) from 30, 50, 70, 80, 90 and 100% at 4 °C for one hour. Then, 100% ethanol was added and samples were kept for another hour at room temperature for complete dehydration. All samples were removed from the ethanol and air-dried under high vacuum (10^−7^ Torr) at room temperature (25 °C) for one day. All dried samples were mounted on an aluminium stub with double-sided adhesive tape (no. 05072-AB; Structure Probe, SPI Supplies, West Chester, PA, USA) and coated with ionic gold (300 A0) in a sputter-coating unit (model E-1010, Hitachi, Japan) at high vacuum. Then, processed samples were scanned under a scanning electron microscope (SEM) (S3400N, Hitachi, Japan) at 15 Kv and high vacuum (10^−7^ Torr), and pictures were taken at different magnifications [29,30].

### 2.7. Immunohistochemical Studies

In this study, 5-HT2A receptor protein immunoreactivity was detected by immunohistochemistry (IHC), using a previously described procedure [31]. Briefly, endogenous peroxidase was blocked by incubation with 3% H_2_O_2_ for 15 min, and sections were incubated overnight at 4 °C in a humidity chamber with primary antibody (1:500 polyclonal anti5-HT2A, catalogue no. MBS175200; MyBioSource, TE Huissen The Netherlands) in blocking solution (3% normal horse serum, VECTASTAIN^®^ Elite^®^ ABC Universal Kit Peroxidase, Horse Anti-Mouse/Rabbit IgG, catalogue no. PK-6200, Burlingame, CA, USA). On the second day, immunostaining was developed using 1:50 biotinylated universal antibody and the avidin/biotin/peroxidase complex amplification system (VECTASTAIN^®^ Elite^®^ ABC Universal Kit Peroxidase (Horse Anti-Mouse/Rabbit IgG, catalogue no. PK-6200)) with the Metal-Enhanced DAB Substrate Kit from Thermo Fisher Scientific, Battle Creek, MI, USA. Since the anti-5-HT2A antibody is a commercial product, a negative control was performed by incubation without primary antibody, and no label was found (not shown).

According to the diffuseness of the staining, sections were graded as 0, <10% or no staining; 1, 10–25% staining; 2, 25–50% staining; 3, 50–75% staining; or 4, >75% staining. According to staining intensity, sections were graded as 0, no staining; 1, weak but detectable staining; 2, distinct staining; or 3, intense staining. Immunohistochemical values were obtained by adding the diffuseness and intensity scores [32].

### 2.8. Statistics

All data were analysed using SPSS Statistics version 19, and the data were represented as mean ± SEM. All behavioural, histopathologic and immunohistochemical evaluations were conducted using the *t*-test as well as two-way ANOVA, performed for pooled analysis between sex differences in this study, at a significance level of *p* < 0.05.

## 3. Results

### 3.1. Physical Appearance

**Figure 2:**
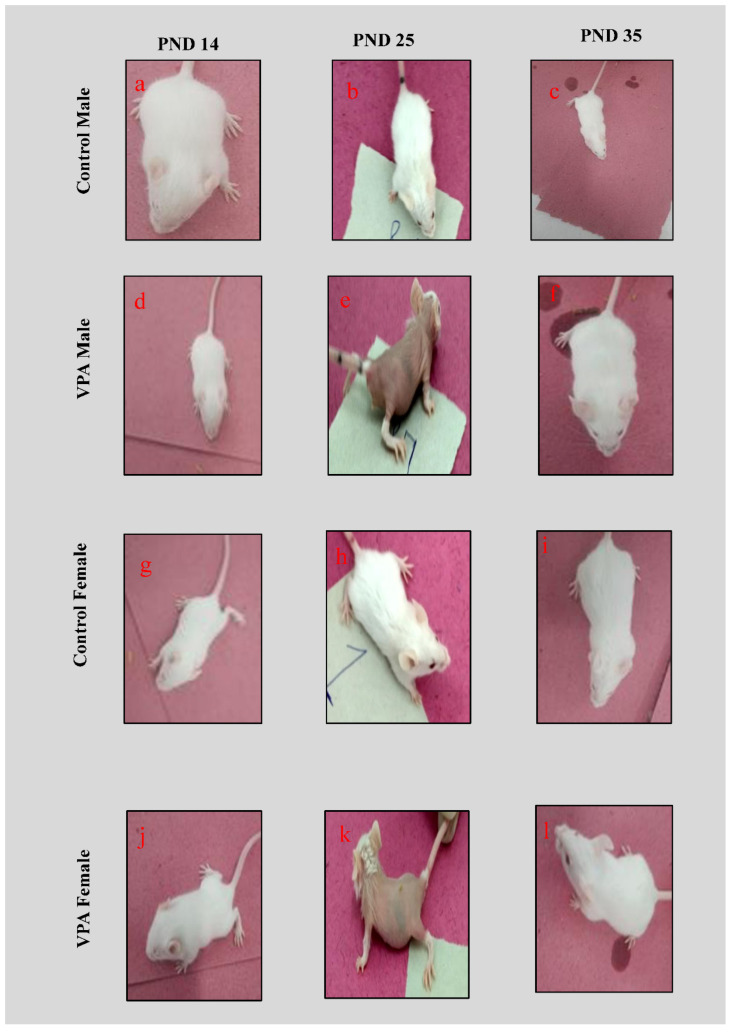
Representative hair coating images from each batch of mice (**a**–**l**) at different age groups (PND 14, 25, 35). Normal hair coating in (**a**–**c**) control male and (**g**–**i**) female. Alopecia was observed in € VPA male and (**k**) female animals.

All control animals showed regular hair coating from PND 14 to 41 (Figure 2a–c,g–i). In contrast, VPA-treated male and female animals showed alopecia from PND 17 to 25 (Figure 2e,k); from PND 28 onwards, hair coating was observed, and animals that reached PND 35 (Figure 2f,l) showed normal hair growth similar to the control.

### 3.2. Balance Beam

The pooled male and female VPA-treated mice showed significantly increased foot slips (*p* < 0.001; Figure 3.1a) compared to the control. There were significant differences in reaching time (*p* < 0.05; Figure 3.1b) and walking length (*p* < 0.05; Figure 3.1c) between the pooled VPA-treated group and control. However, no significant difference was observed in reaching time and walking length between VPA-treated male and female mice as compared to the respective control groups. A significant difference was noticed in foot slips between VPA-treated male (*p* < 0.001) and female (*p* < 0.001) mice as compared to the respective control groups.

### 3.3. Actophotometer

Locomotion was significantly increased in the pooled male and female VPA-treated mice (*p* < 0.001) compared to the control. However, a significant increase in locomotion was observed in male VPA-treated animals (*p* < 0.001) but not female animals (n.s) compared to sex-matched controls, which might be because male animals are more susceptible to VPA. Moreover, there was a significant (*p* < 0.05) difference in locomotion between VPA-treated male and female mice (Figure 3.2), and the locomotion incidence was higher in males (100%) than females (66.6%).

### 3.4. Hot Plate

The nociceptive index was considered to be the time until paw withdrawal in seconds. Our observations revealed a significant increase in the latency of withdrawing the hind paw in the pooled VPA-treated mice compared to the control (*p* < 0.01). There was a significant increase in the latency of withdrawing the hind paw in VPA-treated male animals (*p* < 0.01) but not in females (n.s) compared to sex-matched controls. Furthermore, a significant (*p* < 0.05) difference was noticed in the latency of withdrawing the hind paw between VPA-treated male and female mice (Figure 3.3), and the value was higher for males (66.6%) than females (0%).

### 3.5. Elevated Plus Maze Test

The pooled male and female VPA-treated mice showed a significant decrease in time duration (*p* < 0.001) and number of entries (*p* < 0.001) on the open arms compared to control mice. Significant differences were observed in time duration and number of entries between male (*p* < 0.01, *p* < 0.01) and female (*p* < 0.01, *p* < 0.01) VPA-treated mice compared to sex-matched controls. However, there was no significant difference between VPA-treated male and female mice (Table 1).

### 3.6. Effect on Social Interaction

#### 3.6.1. Social Ability and Social Ability Index

In the social approach test, there was no significant difference in time spent in the stranger chamber between pooled VPA-treated mice and controls (n.s); the former group spent more time in the empty chamber than the latter (*p* < 0.01). However, no significant difference in social ability index between the two groups was observed (*p* < 0.074). There was no significant difference in time spent in the stranger chamber between VPA-treated male and female mice (male, *p* < 0.285; female, *p* < 0.345) compared to sex-matched controls. VPA-treated male mice spent more time (*p* < 0.05) in the empty chamber than controls, but VPA-treated female mice showed no significant difference (*p* < 0.058) compared to controls. VPA-treated male mice a showed significant difference (*p* < 0.05) in the social ability index compared to controls, while female mice did not show any significant differences (*p* < 0.379) (Table 2).

#### 3.6.2. Social Preference and Social Preference Index

In the social preference phase, none of the analysed groups spent significant time in the novel or familiar chamber. Nevertheless, no significant difference was observed in the social preference index among the groups tested (Table 2).

### 3.7. Histopathology of the Brain

In this study, parts of the brain, including the meningeal membranes, cerebral cortex (prefrontal, frontal, and temporal lobe cortex), amygdala, thalamus, ventricles, hippocampus and cerebellum, from male and female animals, were analysed after postnatal VPA exposure.

#### 3.7.1. Cerebral Cortex

Histopathological analysis of the cerebral cortex showed significantly damaged cells in pooled VPA-treated animals (*p* < 0.001) compared to controls. Considerable damage was observed in the frontal and temporal cortex of VPA-treated male mice compared to VPA-treated female mice (Figure 4.1). In contrast to the male mice, in female VPA-treated mice, damaged cells were observed in the prefrontal cortex region (*p* < 0.001), but the remaining cerebral cortex region was intact (Figure 4.1i–p,q).

#### 3.7.2. Hippocampus

As shown in Figure 4.2, there was a significant difference in atrophic cells of the hippocampus in pooled VPA-treated male and female mice (*p* < 0.001) compared to controls. There was a significant increase in atrophic hippocampal cells in VPA-treated male and female mice (both *p* < 0.001) compared to sex-matched controls. However, more atrophic cells were observed in males than females (*p* < 0.05).

#### 3.7.3. Cerebellum

A significant change was observed in atrophic cerebellar Purkinje cells in pooled VPA-treated animals (*p* < 0.001) compared to controls. There was a significant increase in atrophic cerebellar Purkinje cells in VPA-treated males and females (both *p* < 0.001) compared to sex-matched controls. However, no significant changes were observed between males and females (*p* < 0.59) (Figure 4.3).

In this study, histopathological findings of meninges, ventricles, amygdala and thalamus did not show any histological changes in VPA-treated and control mice (data not shown).

### 3.8. Scanning Electron Microscope Analysis

The morphological features of brains from control and VPA-treated mice were assessed using scanning electron microscopy. In our study, three brain regions were selected for SEM analysis: the ultrastructural changes in the peripheral region of the cerebral cortex (Figure 5.1f,n), the hippocampus (Figure 5.1g,o) and the cerebellum (Figure 5.1h,p) were observed in VPA-treated mice compared to control animals. A higher rate of cellular structural changes was observed in VPA-treated males compared to VPA-treated females.

### 3.9. Immunohistochemical Studies

The immunohistochemical evaluation of the cerebral cortex and hippocampus revealed more intense expression of the 5-HT2A receptor protein in VPA-treated male mice compared to control animals and VPA-treated female mice. There was poor or slight expression of the 5-HT2A receptor protein in control animals and VPA females compared with VPA-treated males. Moreover, Purkinje and granular cells showed more intense expression of the 5-HT2A receptor protein in VPA-treated male mice compared with control animals and VPA-treated female mice, and poor or slight expression in control animals and VPA-treated females compared to VPA-treated males (Figure 5.2 and Table 3).

At the end of the behaviour tests, histopathology analysis and immunohistochemical studies, and from the critical analysis of the data generated, we could conclude that male mice were more susceptible to VPA treatment than females.

## 4. Discussion

In the present study, we examined behavioural deficits, brain histopathological changes and 5-HT2A receptor protein expression in male and female animals with VPA-induced autism. BALB/c mice were chosen, as these animals are less reactive to social contact in VPA-induced autism than many other inbred strains, including C57/129 mice [33]. In line with previous reports, in the current study, administration of sodium valproate to PND 14 mice resulted in autistic symptoms such as decreased pain sensitivity, loss of motor skill development (balance beam test), increased locomotor activity, social behaviour deficits and increased anxiety in the elevated plus maze [9,12,14,26]. There was a higher trend of ASD incidence in male mice compared to female mice in this study, which is in line with a behavioural-based study in humans that suggested a higher incidence of ASD (2 to 3 times) in men than women [12].

In this study, for the first time, we observed that VPA-treated mice showed hair loss from PND 17 to 25, which might be due to a biotin deficiency [34,35]; from PND 28 onwards, hair growth was observed, and by the time animals reached PND 35, regular hair coating was noticed that was similar to control animals. In the balance beam test, foot slip changes were observed in VPA-treated male and female mice that may have been due to the altered histoarchitecture of the cerebral cortex, which was pronounced in males. The cerebral cortex, deep cerebellar nuclei and striatum play pivotal roles in movement coordination [27].

In the current study, male VPA mice exhibited enhanced locomotor activity compared to females, which is mainly due to the hyperactivity/hyperexcitability caused by increased glutaminergic transmission [9]. Previous reports have also suggested abnormalities in the GABAnergic system in epilepsy and ASD [36]. Excess glutamate release and absorption have been observed in brain regions such as the frontal cortex and hippocampus of rats exposed to different levels of stress, indicating that glutamate excitotoxicity is related to extreme stress [37] and leads to hippocampal abnormality [38].

In this study, we observed increased latency of withdrawing the hind paw in VPA-treated male animals than VPA-treated females. Sensory pathways are networks of neurons that run from the sensory organ to the cerebral cortex, and they are responsible for sensation perception [39]. We observed that the whole cerebral cortex region was damaged in VPA-treated male animals, but found damaged cells only in the prefrontal cortex in VPA-treated female mice. This may be the reason for the increased nociceptive threshold in VPA-treated male animals.

The elevated plus maze test was conducted to determine the level of fear and anxiety. VPA-treated male mice showed less anxiety compared to VPA-treated female mice, which may reflect anxiety and typical forms of depression among women [40]. Contrary to previous reports, problems with anxiety and repetitive behaviour were observed in male rats treated with VPA [41]. In accordance with previous studies, VPA-treated animals in our study exhibited damaged cells in the prefrontal cortex region, which may have been a cause of the elevated anxiety levels in the animals [13].

Social behaviour is a primary deficit in ASD [12]. In the social approach test, from PND 30 to 40, differences between saline- and VPA-treated mice in their willingness to approach a social target were not seen. We investigated sociability, social index, social preference and social preference index in the animals and found that VPA-treated male and female mice did not show any significant reduction in the duration of time spent in the stranger chamber, but males spent more time than females in the empty chamber. However, postnatal VPA-treated male mice displayed a significant decrease only in the social index parameter, which is again contradictory to previous studies that showed low sociability and a reduction in social index in VPA-treated mice [12]. Furthermore, previous studies reported no differences in the social approach test between saline- and VPA-treated mice [42]. However, Bertolino et al. [12] and Norton et al. [42] used C57BL/6 mice, whereas, in this study, we used BALB/c mice. In agreement with previous results, in the social preference phase, postnatal VPA-treated male and female mice did not show significantly low social preference or social preference index [42].

In this investigation, we exposed PND 14 mice to VPA because this stage of mouse development approximately corresponds to the third trimester in human development, bringing about changes in different parts of the whole brain. In the present study, we observed that both male and female mice showed similar morphological changes in the prefrontal cortex and Purkinje cells. However, more changes were observed in the cerebral cortex, including the frontal cortex temporal lobe, and the hippocampus in VPA-treated male but not female mice. Nonetheless, detailed morphological observation using scanning electron microscopy (SEM) showed a higher rate of structural changes in male VPA animals. The structural changes may have caused social and behavioural alterations in these animals. Furthermore, histopathological analysis of meninges, ventricles, amygdala and thalamus did not show any histological changes in both VPA-treated and control mice. A few clinical reports suggested that there is an altered histoarchitecture of meninges in autistic patients [43,44].

Cerebellum and hippocampus abnormalities have been found to be important in the pathophysiology of autism disorder due to their active role in the learning process, motor functions, anxiety, social functioning and emotions, which are primarily altered in autism [13,45]. Purkinje cells and granules are the two major neuronal circuits in the cerebellum [26]. Purkinje cells represent major cerebellar connections with the cerebral cortex and limbic system, and, during the postnatal period, they are postmitotic [9,46]. Studies on neuropathology have shown that abnormalities related to the organisation of the cytoarchitecture of the cerebral cortex and subcortical structures with decreased Purkinje cell numbers in the cerebellum are the most common histopathological findings in the brain tissue of autistic patients. These findings suggest that disorders in cortical organisation and neuronal maturation could be the root cause of neurological problems in patients suffering from autism [47]. In our study, more cerebral cortical changes were observed in the brains of VPA-treated male mice than female mice, and this may be reason for the social and behavioural deficits in male animals. The main reason that the male brain is sensitised to autism is variations in the concentrations of hormones such as androgen and oestrogen in the brain, causing a bias (androgens are converted to oestrogens in the brain via aromatase, and males have higher oestrogen concentrations). These hormones are powerful behaviour controllers. Sex steroids may affect the excitatory/inhibitory balance in the male brain, making it more susceptible to ASD [48].

There is substantial evidence that the 5-HT2A receptor plays a major role in causing the neurophysiological effects of serotonergic hallucinogens in experimental animals as well as human subjects [49]. In agreement with previous reports, in our study, VPA-exposed male animals showed upregulated 5-HT2A receptor expression compared to VPA-treated female animals [50], which was inconsistent with other clinical reports [51]. Moreover, the 5-HT2A receptor is responsible for hallucinogenic activity and plays a vital role in the process of learning and memory. In addition, it has also been shown to be a dominant aspect in many psychological disorders, such as schizophrenia, depression, obsessive-compulsive disorder and autism [23].

In brief, according to previous reports, the effect on postnatal behaviour is similar in both male and female offspring of VPA animals, but social behavioural changes are confined to VPA-treated male mice [19]. In contrast to a study by Kazlauskas et al. [19], female mice in the experimental group did not show any behavioural deficits, such as in locomotion or nociception activity, as male animals did [19]; however, the aforementioned authors used outbred CrlFcen:CF1 mice, whereas we used BALB/c mice. We observed that the number of damaged brain cells and downregulation of 5-HT2A receptor protein expression in the offspring of VPA-treated females decreased considerably compared to males, and this may have been the cause of the social and behavioural deficits in males. We observed these differences in male offspring. Future studies in this field should evaluate whether these differences are confined to the juvenile stage or can be found in adult animals too. In summary, our study suggests that VPA-treated male animals are affected by behavioural deficits more than females.

## 5. Conclusions

In conclusion, to the best of our knowledge, the present study demonstrates that inducing autism by VPA brings about substantial differences in the brain histopathology of both male and female BALB/c mice, characterised by changes in the cerebral cortex, frontal cortex and temporal lobe in VPA-treated male animals only and changes in the prefrontal cortex, hippocampus and Purkinje cells in all VPA-treated animals. For the first time, we observed hair loss from PND 17 to 25 in a VPA-induced autism animal model. A higher rate of cellular structural changes in the brain was observed in VPA-treated male mice. Moreover, upregulation of 5-HT2A receptor protein expression was also observed in VPA-treated male mice. These findings suggest that VPA-induced morphological abnormalities and 5-HT2A receptor protein expression in the cerebral cortex, hippocampus and cerebellum may be involved in sex-dependent behavioural and social index deficits. The present study demonstrates that VPA treatment can induce autism in male BALB/c mice, and this can be an animal model for further autism studies.

## Figures and Tables

**Figure 3:**
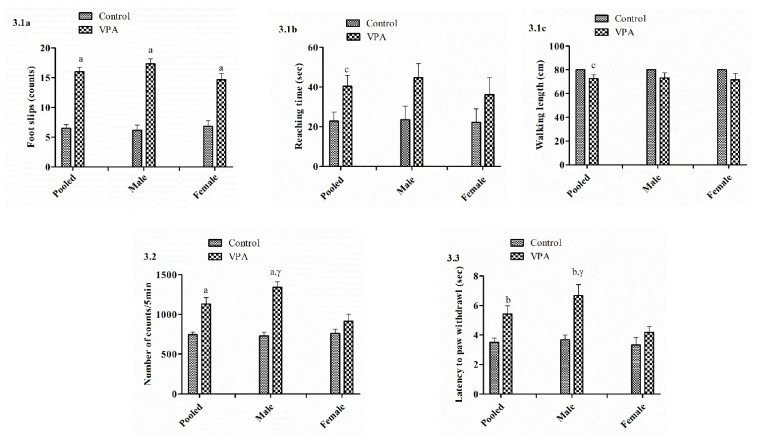
(**3.1**) Motor coordination ability of male and female BALB/c mice following administration of sodium valproate (VPA, 400 mg/kg s.c.) was measured using the balance beam test on postnatal day 25: (**a**) foot slips, (**b**) reaching time (s), (**c**) walking length. (**3.2**) Locomotion due to hyperactivity measured in male and female BALB/c mice following administration of sodium valproate (VPA, 400 mg/kg s.c.) using the actophotometer on postnatal day 35. (**3.3**) Nociceptive response of male and female BALB/c mice following administration of sodium valproate (VPA, 400 mg/kg s.c.) was assessed by using Eddy’s hot plate on postnatal day 38. Data expressed as mean ± SEM. Pooled, *n* = 12; males, *n* = 6; females, *n* = 6. ^a^ *p* < 0.001, ^b^ *p* < 0.01, ^c^ *p* < 0.05 vs. sex-matched control group; ^α^ *p* < 0.001, ^β^ *p* < 0.01, ^γ^ *p* < 0.05 vs. VPA-treated female mice (using *t*-test), n.s, non-significant (*p* > 0.05).

**Figure 4:**
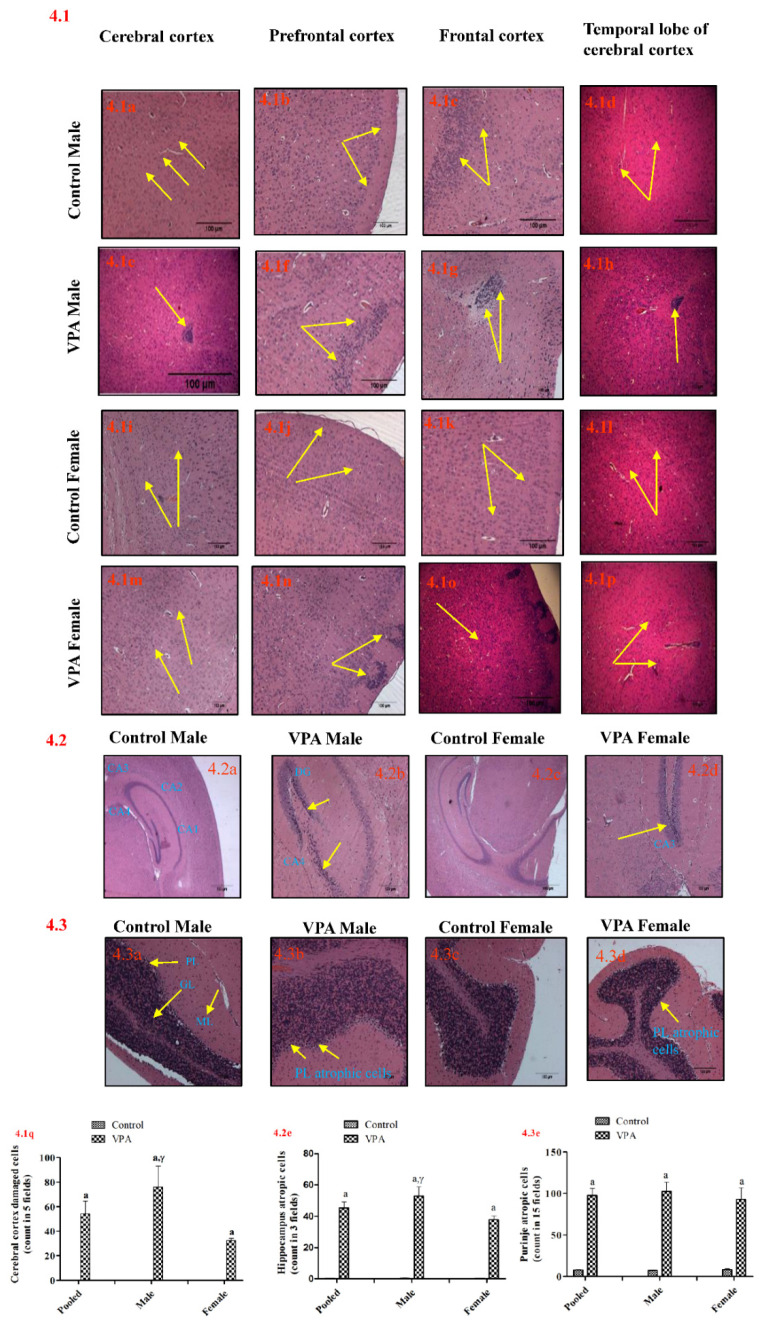
(**4.1**) Photomicrographs of sections of cerebral cortex. (**a**–**d**) Control male animals showed normal neurons in cerebral, prefrontal, frontal and temporal cortex. VPA male animals showed (**e**) chronic inflammatory cells in cerebral cortex, (**f**) degenerated or apoptotic neurons in prefrontal cortex, (**g**) inflammation in frontal cortex and (**h**) inflammatory cells in temporal lobe of cerebral cortex. (**i**–**l**) Control female animals showed normal neurons in cerebral, prefrontal, frontal and temporal cortex. VPA female animals showed (**m**,**o**,**p**) normal neurons in cerebral, frontal and temporal cortex and (**n**) focal gliosis in prefrontal cortex. (**q**) Number of cerebral cortex damaged cells in sex-matched VPA-treated animals compared to sex-matched controls. Damaged cerebral cortex neurons were counted with 5-field analysis using a microscope. (**4.2**) Photomicrographs of hippocampus. (**a**,**c**) Sex-matched control groups showed normal hippocampal neurons; (**b**) atrophic neurons in hippocampus of VPA-treated male animals; (**d**) atrophic neurons in hippocampus of VPA-treated female animals; CA, Cornu Ammonis; DG, Dentate gyrus; (**e**) number of atrophic hippocampal cells in sex-matched VPA-treated animals compared to sex-matched controls. Damaged hippocampal neurons were counted with 3-field analysis using a microscope. (**4.3**) Photomicrographs of sections of cerebellum. (**a**,**c**) Sex-matched control animals showed normal Purkinje layer; (**b**) atrophic Purkinje neurons in VPA-treated male animals; (**d**) atrophic Purkinje neurons in VPA-treated female animals. PL, Purkinje layer; ML, molecular layer; GL, granular layer; (**e**) atrophic Purkinje cells in sex-matched VPA-treated animals compared to sex-matched controls. Damaged Purkinje neurons were counted with 15-field analysis using a microscope. Scale bar: 100 µm. Final magnification 10×. Data expressed as mean ± SEM. Pooled, *n* = 12; males, *n* = 6; females, *n* = 6. ^a^ *p* < 0.001, ^b^ *p* < 0.01, ^c^ *p* < 0.05 vs. sex-matched controls; ^α^ *p* < 0.001, ^β^ *p* < 0.01, ^γ^ *p* < 0.05 vs. VPA-treated female mice (using *t*-test), n.s, non-significant (*p* > 0.05).

**Figure 5:**
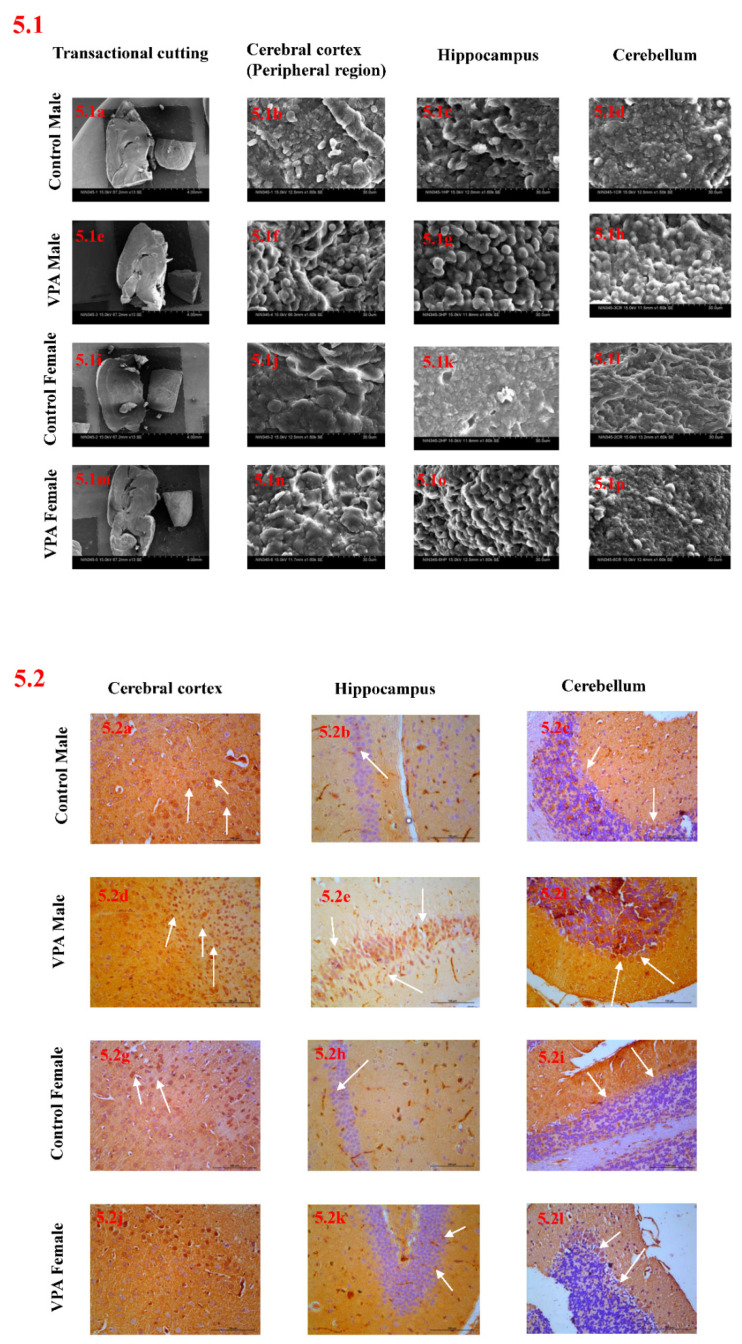
(**5.1**) Scanning electron microscopy photomicrographs of brain: (**a**,**i**) transactional cutting of brains of sex-matched control animals; (**e**,**m**) transactional cutting of brains of sex-matched VPA animals; (**b**,**j**) peripheral region of cerebral cortex of sex-matched control animals; (**f**,**n**) peripheral region of cerebral cortex of sex-matched VPA animals; (**c**,**k**) hippocampus of sex-matched control animals; (**g**,**o**) hippocampus of sex-matched VPA animals; (**d**,**l**) cerebellum of sex-matched control animals; (**h**,**p**) cerebellum of sex-matched VPA animals. Scale bar: 30 µm. (**5.2**) Effect of postnatal exposure to VPA on immunoreactivity of 5-HT2A receptor in mouse brain. Representative microphotography showing immunoexpression of 5-HT2A receptor protein in cerebral cortex, hippocampus and Purkinje layer of (**a**–**c**) control male mice, (**d**–**f**) VPA male mice, (**g**–**i**) control female mice and (**j**–**l**) VPA female mice. Scale bar: 100 µm; final magnification 40×. 5-HT2A receptor protein expression (white arrows). Data expressed as mean ± SEM. Pooled, *n* = 6; males, *n* = 3; females, *n* = 3. ^a^ *p* < 0.001, ^b^ *p* < 0.01, ^c^ *p* < 0.05 vs. sex-matched controls; ^α^ *p* < 0.001, ^β^ *p* < 0.01, ^γ^ *p* < 0.05 vs. VPA-treated female mice (using *t*-test). n.s, non-significant (*p* > 0.05).

**Table 1:** Effect of VPA on anxiety using elevated plus maze apparatus.

	Male			Female			Pooled		
Parameters	Control	VPA	*p*-Value	Control	VPA	*p*-Value	Control	VPA	*p*-Value
No. of entries in open arms	10.17 (2.02)	3.17 (0.91) ^b^	0.01	6.67 (0.95)	2.00 (0.58) ^b^	0.002	8.42 (1.19)	2.58 (0.54) ^a^	0.001
Time spent in open arms (s)	97.17 (9.25)	46.17 (12.08) ^c^	0.007	77.17 (10.42)	25.83 (7.39) ^b^	0.002	87.17 (7.29)	36.00 (7.41) ^a^	0.001

Data expressed as mean ± SEM. *n* = 12 per group (6 males and 6 females). ^a^ *p* < 0.001, ^b^ *p* < 0.01 and ^c^ *p* < 0.05 vs. sex matched control group; ^α^ *p* < 0.001, ^β^ *p* < 0.01 and ^γ^ *p* < 0.05 vs. when compared to VPA-treated female mice (using *t*-test). n.s, non-significant (*p* > 0.05).

**Table 2:** Effect of VPA on sociability and sociability index; social preference and social preference index determined using three-chamber social interaction testing apparatus.

	Male			Female			Pooled		
Parameters	Control	VPA	*p*-Value	Control	VPA	*p*-Value	Control	VPA	*p*-Value
Time spent in stranger chamber	313.67 (17.03)	276.83 (27.80)	0.285	300.83 (35.19)	236.67 (54.31)	0.345	307.25 (18.7)	256.75 (29.71)	0.165
Time spent in empty chamber	166.67 (12.82)	236.83 (19.38) ^b^	0.013	207.83 (26.75)	311.33 (40.28)	0.058	187.25 (15.45)	274.08 (24.09) ^b^	0.006
Social ability index	1.93 (0.16)	1.24 (0.21) ^c^	0.026	1.70 (0.44)	1.08 (0.51)	0.379	1.82 (0.23)	1.16 (0.27)	0.074
Time spent in novel animal	280.00 (17.83)	258.00 (17.28)	0.396	305.50 (35.25)	288.83 (47.61)	0.784	292.75 (19.22)	273.42 (24.59)	0.542
Time spent in familiar animal	218.17 (22.82)	247.83 (16.42)	0.316	220.17 (29.61)	237.50 (46.05)	0.758	219.17 (17.82)	242.67 (23.36)	0.432
Social preference index	1.38 (0.20)	1.07 (0.11)	0.215	1.64 (0.42)	1.66 (0.53)	0.98	1.51 (0.23)	1.37 (0.27)	0.687

Data expressed as mean ± SEM. *n* = 12 per group (6 males and 6 females). ^a^ *p* < 0.001, ^b^ *p* < 0.01 and ^c^ *p* < 0.05 vs. sex matched control group; ^α^ *p* < 0.001, ^β^ *p* < 0.01 and ^γ^ *p* < 0.05 vs. when compared to VPA-treated female mice (using *t*-test). n.s, non-significant (*p* > 0.05).

**Table 3:** The immunohistochemical staining scores of sex differences in control and VPA groups.

	Male			Female			Pooled		
Parameters	Control	VPA	*p*-Value	Control	VPA	*p*-Value	Control	VPA	*p*-Value
Cerebral cortex	2.33 (0.33)	4.00 (0.00) ^c,γ^	0.03	2.00 (0.00)	2.67 (0.33)	0.11	2.17 (0.17)	3.33 (0.33) ^c^	0.02
Hippocampus	1.00 (0.00)	3.67 (0.3) ^c,γ^	0.03	1.00 (0.00)	1.00 (0.00)	1.00	1.00 (0.00)	2.33 (0.61)	0.06
Purkinje cells	2.00 (0.00)	4.67 (0.33) ^c,γ^	0.03	2.00 (0.00)	3.00 (0.00) ^c^	0.02	2.00 (0.00)	3.83 (0.40) ^b^	0.002

Data expressed as mean ± SEM. *n* = 6 per group (3 males and 3 females). ^a^ *p* < 0.001, ^b^ *p* < 0.01 and ^c^ *p* < 0.05 vs. sex matched control group; ^α^ *p* < 0.001, ^β^ *p* < 0.01 and ^γ^ *p* < 0.05 vs. when compared to VPA-treated female mice (using *t*-test). n.s., non-significant (*p* > 0.05).

## Data Availability

Not applicable.
